# Genome-wide response on phytosterol in 9-hydroxyandrostenedione-producing strain of *Mycobacterium* sp. VKM Ac-1817D

**DOI:** 10.1186/s12896-019-0533-7

**Published:** 2019-06-25

**Authors:** Eugeny Y. Bragin, Victoria Y. Shtratnikova, Mikhail I. Schelkunov, Dmitry V. Dovbnya, Marina V. Donova

**Affiliations:** 1 Institute of Biochemistry and Physiology of Microorganisms, Federal Research Center “Pushchino Center for Biological Research of the Russian Academy of Sciences”, Nauki, 5, Pushchino, Russian Federation 142290; 2 Pharmins Ltd., Institutskaya, 4, Pushchino, Russian Federation 142290; 3 Skolkovo Institute of Science and Technology, Nobelya, 3, Moscow, Russian Federation 121205; 4 A.N. Belozersky Research Institute of Physico-Chemical Biology, M.V. Lomonosov Moscow State University, Leninskye gory, 1, building 40, Moscow, Russian Federation 119992; 5 Institute for Information Transmission Problems, Russian Academy of Sciences, Bolshoy Karetny, 19, build. 1, Moscow, Russian Federation 127051

**Keywords:** Steroid catabolism, Bioconversion, Phytosterol, 9α-hydroxyandrostenedione, *Mycobacterium*, Transcriptome

## Abstract

**Background:**

Aerobic side chain degradation of phytosterols by actinobacteria is the basis for the industrial production of androstane steroids which are the starting materials for the synthesis of steroid hormones. A native strain of *Mycobacterium* sp. VKM Ac-1817D effectively produces 9α-hydroxyandrost-4-ene-3,17-dione (9-OH-AD) from phytosterol, but also is capable of slow steroid core degradation. However, the set of the genes with products that are involved in phytosterol oxidation, their organisation and regulation remain poorly understood.

**Results:**

High-throughput sequencing of the global transcriptomes of the *Mycobacterium* sp. VKM Ac-1817D cultures grown with or without phytosterol was carried out. In the presence of phytosterol, the expression of 260 genes including those related to steroid catabolism pathways significantly increased. Two of the five genes encoding the oxygenase unit of 3-ketosteroid-9α-hydroxylase (*kshA*) were highly up-regulated in response to phytosterol (55- and 25-fold, respectively) as well as one of the two genes encoding its reductase subunit (*kshB*) (40-fold). Only one of the five putative genes encoding 3-ketosteroid-∆^1^-dehydrogenase (KstD_1) was up-regulated in the presence of phytosterol (61-fold), but several substitutions in the conservative positions of its product were revealed.

Among the genes over-expressed in the presence of phytosterol, several dozen genes did not possess binding sites for the known regulatory factors of steroid catabolism. In the promoter regions of these genes, a regularly occurring palindromic motif was revealed. The orthologue of TetR-family transcription regulator gene *Rv0767c* of *M. tuberculosis* was identified in *Mycobacterium* sp. VKM Ac-1817D as *G155_05115*.

**Conclusions:**

High expression levels of the genes related to the sterol side chain degradation and steroid 9α-hydroxylation in combination with possible defects in KstD_1 may contribute to effective 9α-hydroxyandrost-4-ene-3,17-dione accumulation from phytosterol provided by this biotechnologically relevant strain. The TetR-family transcription regulator gene *G155_05115* presumably associated with the regulation of steroid catabolism. The results are of significance for the improvement of biocatalytic features of the microbial strains for the steroid industry.

**Electronic supplementary material:**

The online version of this article (10.1186/s12896-019-0533-7) contains supplementary material, which is available to authorized users.

## Background

Steroids are terpenoid lipids that contain a gonane core of four cycloalkane rings (A-D) in their structure. This class of organic molecules includes the compounds which fulfil essential vital functions in higher organisms. Sterols such as cholesterol, β-sitosterol, stigmasterol, campesterol etc. are steroid 3β-alcohols with different aliphatic side chains at C17.

The molecular mechanisms of sterol degradation have been intensively studied, mainly due to their essential role in the pathogenesis of *Mycobacterium tuberculosis* [1, 2] and the wide application of non-pathogenic mycobacteria capable of partial catabolism of sterols to produce intermediates for the pharmaceutical industry, such as androst-4-ene-3,17-dione (AD), androsta-1,4-diene-3,17-dione (ADD) and 9α-hydroxyandrost-4-ene-3,17-dione (9-OH-AD). 9-OH-AD is extensively used as a starting compound for the synthesis of various steroid drugs bearing a halogen in position 9, such as dexamethasone, fluoxymesterone, triamcinolone, bethametasone and others [3].

**Fig. 1:**
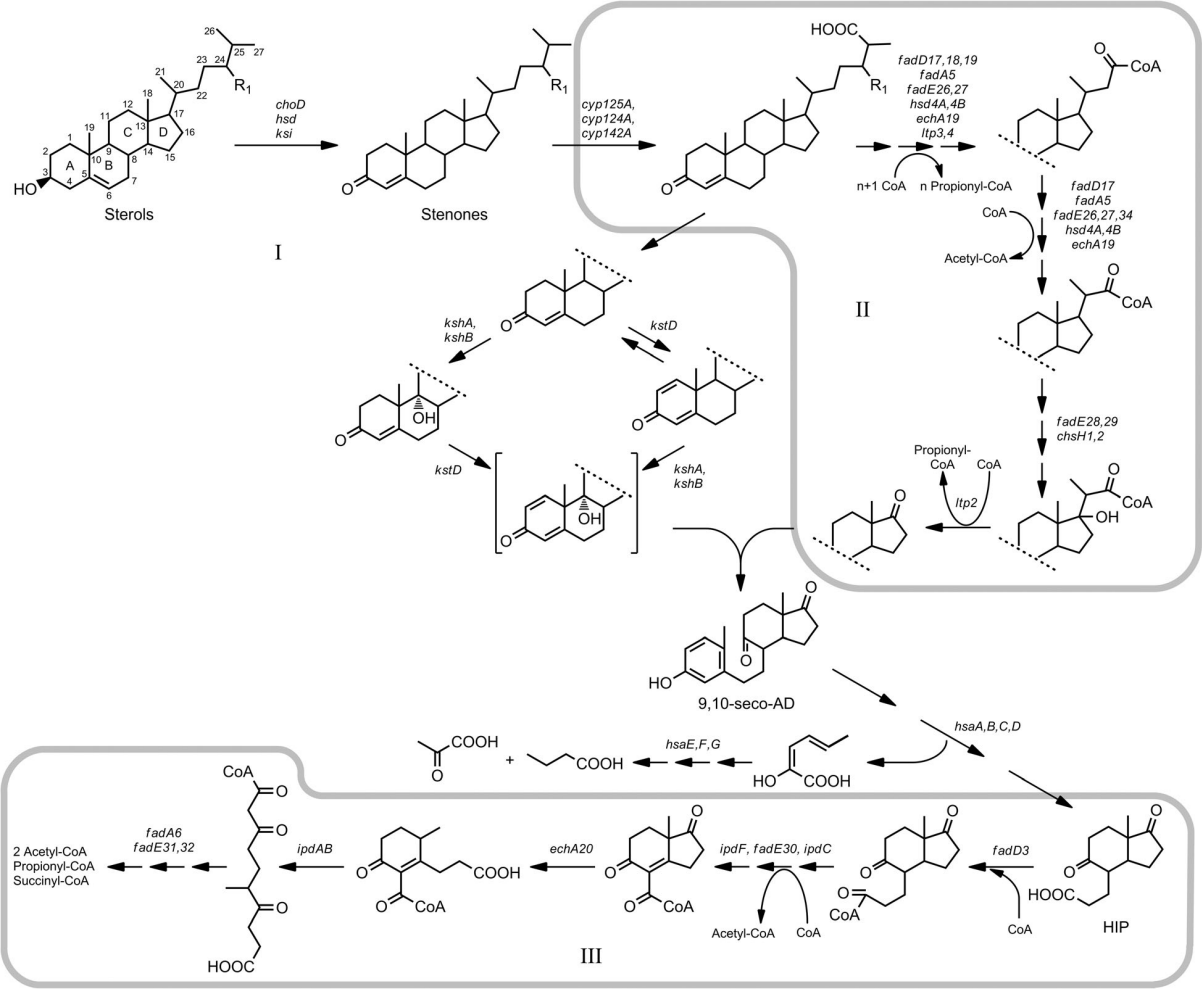
General scheme of sterol catabolism organisation in Actinobacteria: stages and the identified genes involved. I – initial steroid core oxidation (rings A-B), II – side chain degradation, III – deep steroid core oxidation (rings C-D). HIP – 3aα-H-4α(3′-propanoate)-7aβ-methylhexahydro-1,5-indanedione; 9,10-seco-AD – 3-hydroxy-9,10-secoandrost-1,3,5(10)-triene-9,17-dione; COCHEA-CoA – 2-(2-carboxyethyl)-3-methyl-6-oxocyclohex-1-ene-1-carboxyl-CoA; R1 – H, CH3 or C2H5 for various sterols

Steroid-degrading bacteria are widespread in the environment, and play an important role in the global carbon cycle [4]. Bacterial sterol degradation represents cascades of reactions which can be conventionally divided according to the parts of the steroid molecule: aliphatic side chain, rings A/B and rings С/D degradation (Fig. 1). Dozens of enzymes are involved in this process. The groups of putative genes engaged in sterols degradation have been characterised in the genomes of several strains such as *Mycobacterium neoaurum* VKM Ac-1815D, *Gordonia neofelifaecis* NRRL B-59395, *M. tuberculosis* H37Rv*, Rhodococcus jostii* RHA1, *M. neoaurum* NRRL 3805B, *Nocardioides simplex* VKM Ac-2033D and *M. smegmatis* mc^2^ 155 [2, 5–9]. It had been proposed that steroid catabolism pathways were conserved in certain different *Actinobacteria* taxa [10].

The first reaction of the pathway is modification of the 3β-hydroxy-5-ene to 3-keto-4-ene- moiety (Fig. 1). Cholesterol oxidases (ChOs) and 3β-hydroxysteroid dehydrogenases (3-HsDs) are able to carry out this reaction (for review, see [11]). In some strains leading role is played by ChOs [12], while in several mycobacteria species the reaction was proposed to be mainly catalysed by 3-HsDs [9, 13]. Side chain degradation is initiated with hydroxylation at C-26(27) with cytochrome P450 monooxygenase encoded by *cyp125* [14, 15]. Similar activity has been confirmed for the proteins encoded by *cyp124* and *суp142* [16]. Degradation of the C24-branched chain sterols was shown to occur via the aldolytic cleavage with aldol lyases encoded by *ltp3* and *ltp4* [17].

The pathway of the following side chain degradation of sterols is generally similar to the fatty acids β-oxidation. CoA is attached to the carboxyl group of cholestenoate with acyl-CoA synthetase encoded by *fadD19* [18]. The CoA-thioester formed is further subjected to three successive cycles of β-oxidation to complete the degradation of the side chain, resulting in the production of C17-keto androstanes [14, 19, 20].

Steroid core degradation proceeds through the well-known “9(10)-seco pathway” with the key steps catalysed by 9α-hydroxylase (Ksh) and 3-ketosteroid-∆^1^-dehydrogenase (KstD) [21, 22]. Their cooperative action results in the ring B opening to form 3-hydroxy-9,10-seconandrost-1,3,5(10)-triene-9,17-dione (3-HSA) (Fig. 1). Metabolic blocks preventing activity of KstD and/or Ksh may provide effective accumulation of the useful intermediates such as AD, ADD, or 9-OH-AD [1, 23, 24].

Further rings A/B degradation is catalysed by the enzymes encoded by *hsaA*, *hsaB*, *hsaC*, *hsaD*, *hsaE*, *hsaF*, *hsaG* [25–27]. Most of the genes coding for the enzymes of the side chain and A/B rings degradation are regulated with KstR transcription factor [28].

As has been proven for several actinobacteria strains, the side chain and rings A/B degradation may occur simultaneously [21, 22, 29]. Moreover, some cholesterol ring-degrading enzymes were shown to exhibit much higher activities towards the side-chain degradation intermediates than towards the corresponding C-17 ketosteroids with fully degraded side chain, e.g. steroid CoA-thioesters represent physiological substrates for Ksh of *M. tuberculosis* [21]. These results were further confirmed for *M. neoaurum* ATCC 25795: greater activities of Ksh (KshA1, KshA2) have been demonstrated in vitro towards 23,24-bisnorchol-1,4-diene-22-oic acid (1,4-BNC) than towards AD [24, 30]. It coincides with the structural features of KshA (for review, see [31]) [21, 32] and the resulting transcriptional changes of *kshA1* and *kshA2*: unlike cholesterol, AD was shown to be a poor inducer for both enzymes in *M. neoaurum* ATCC 25795 [30]. At the same time, KshAs demonstrate lower activity towards cholestenone than towards C19-steroids such as AD, ADD, or testosterone [30, 33].

The KstD enzyme may also be involved in different stages of steroid catabolism, in particular, KstD activity has been identified for both AD and partially oxidised side chain steroids, such as 22-hydroxy-23,24-bisnorchola-4-en-3-one [34].

The C/D rings of the steroid core are degraded by the products of the genes regulated with KstR2. KstR2 is a transcription repressor which similar to KstR belongs to TetR family. As previously shown, the effector of KstR2 is HIP-CoA which is a thioester of cholesterol metabolite HIP (3aα-*H*-4α(3-propanoate)-7aβ-methylhexahydro-1,5-indanedione) [35]. Regulon KstR2 in *M. smegmatis* mc^2^ 155 was reported to consist of 15 genes (*MSMEG_5999*—*MSMEG_6004*, *MSMEG_6008*, *MSMEG_6009*, *MSMEG_6011*—*MSMEG_6017*) including *kstR2* itself (*MSMEG_6009*) [36]. For many genes of the KstR2 regulon, the participation in HIP catabolism has been already proven [37–39], while the functions of some genes of this regulon have not been cleared yet.

Whole transcriptome analysis is being applied increasingly more often in sterol biodegradation research in order to reveal the complete set of the genes whose expression is induced in the presence of sterols, such as cholesterol or phytosterols [2, 9, 40, 41]. This approach allows the identification of those genes whose products are directly or indirectly involved in the sterol degradation pathways including the genes that have not been known before as related to sterol catabolism. Moreover, this method makes it possible to determine how the specific genes are grouped within the genome, as well as to estimate the features of their regulation.

One of the first investigations which exploited whole genome approach showed that 572 genes in *R. jostii* RHA1 increased the level of their expression more than 2-fold in the presence of cholesterol. These genes were grouped into 6 clusters, but only two of them included the known genes related to sterol catabolism [2]. In *M. smegmatis* mc^2^ 155, the expression of 89 genes increased more than 3-fold in response to cholesterol [9]. Most of these genes were grouped into 3 clusters including the genes involved in sterol catabolism. Study of the global transcriptome of *M. smegmatis* mc^2^ 155 unraveled 454 genes increased their expression in the presence of cholesterol as compared to the control. Eleven and sixteen gene clusters were induced by cholesterol when compared with glycerol, or androstenedione, respectively [40]. However, in spite of the growing body of research on bacterial steroid degradation, many aspects of the catabolic pathways regulation remain unknown, especially those related to phytosterol oxidation by saprotrophic fast-growing mycobacteria which were recently suggested to re-classify as *Mycolicibacterium* in accordance with modern taxonomy [42].

Unlike other cholesterol-degrading or phytosterol-transforming mycobacteria such as *M. tuberculosis*, *M. smegmatis* mc^2^ 155, *M. neoaurum* VKM Ac-1815D, VKM Ac-1816D, NRRL 3805B or NRRL 3683B, the saprotrophic fast-growing wild-type strain of *Mycobacterium* sp. VKM Ac-1817D effectively produces 9-OH-AD from phytosterol [5, 23]. The identification of side-chain degradation intermediates showed the presence of 9α-hydroxyl function in their structures, thus suggesting the action of Ksh at the early stages of phytosterol degradation [23]. However, effective 9-OH-AD accumulation by this strain was shown to be accompanied with slow steroid degradation due to residual 3-ketosteroid-Δ^1^-dehydrogenase activity [43].

Earlier, we had carried out a comparative study of the sequences of steroid catabolism genes in the whole genome scales in *Mycobacterium* sp. VKM Ac-1817D (*Myc* 1817) and two *M. neoaurum* strains, namely, VKM Ac-1815D and 1816D converting phytosterols to AD and ADD, respectively [5]. The complete genome of *Myc* 1817 was later assembled [44]. In comparison with *M. neoaurum*, *Myc* 1817 possesses a larger genome (6.35 Mbp) containing more homologues of the genes with putative role in sterol catabolism, including several copies of *kshA*, *kshB* and *kstD*. Based on 16S rRNA phylogeny, *Myc* 1817 is in the close relationship with *M. gilvum* and *M. smegmatis* [5]. These data, in combination with specific catalytic features of *Myc* 1817D strain, and mainly the ability for 9α-hydroxylation of all the intermediates of sterol side chain degradation with accumulation of 9-OH-AD as a major product, allowed us to predict the differences in the induction pattern.

In this study, we estimated genome-wide response on phytosterol in *Mycobacterium* sp. VKM Ac-1817D based on RNA-sequencing and whole-transcriptome analysis in order to understand peculiar properties of this organism.

## Results

### Growth and phytosterol bioconversion

**Fig. 2:**
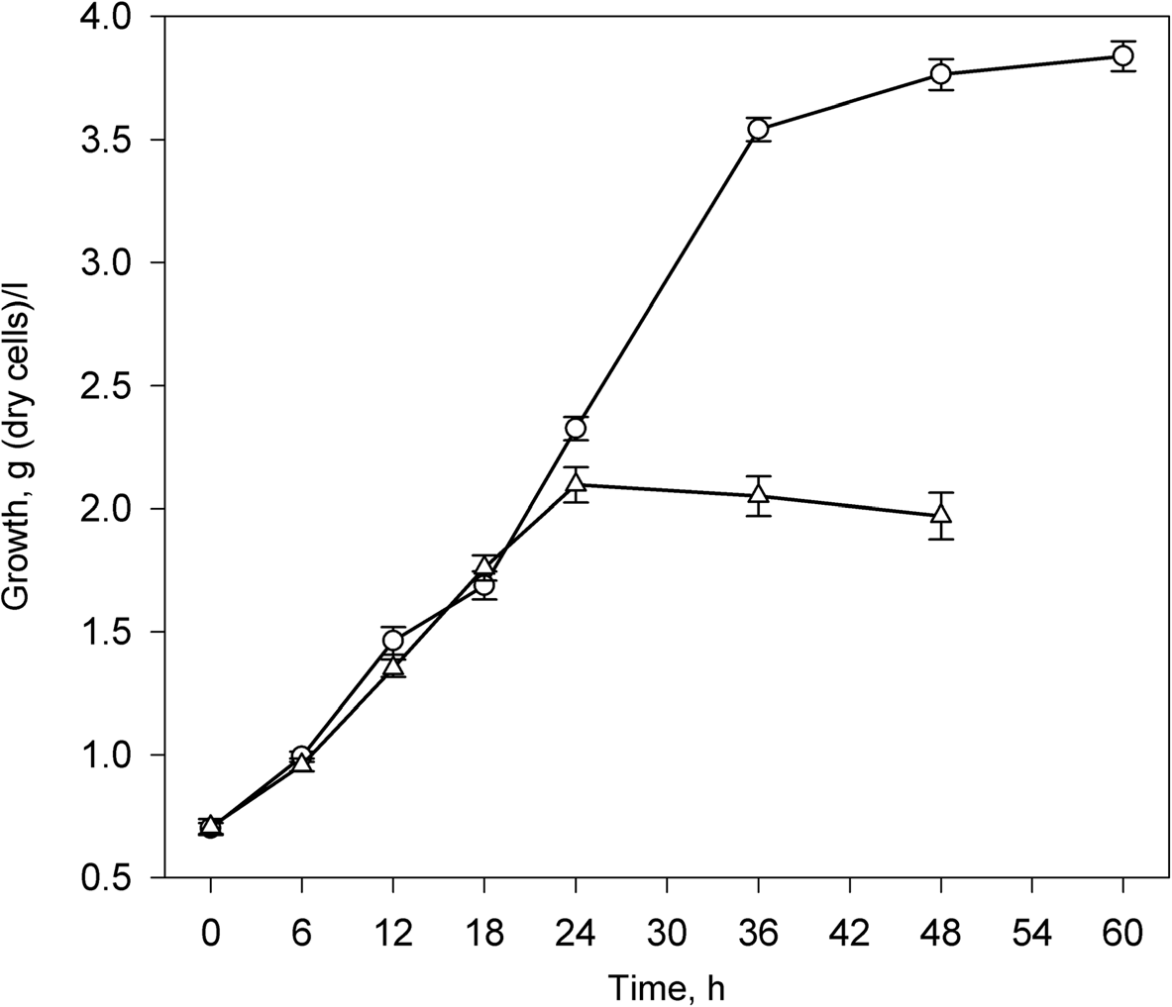
Growth of *Mycobacterium* sp. VKM Ac-1817D on the control medium with 5 g/l glycerol as the carbon source (triangles) and induction medium supplemented with 12 mmol/l phytosterol (circles). The values represent the average of at least three independent experiments; error bars represent the standard deviations

**Fig. 3:**
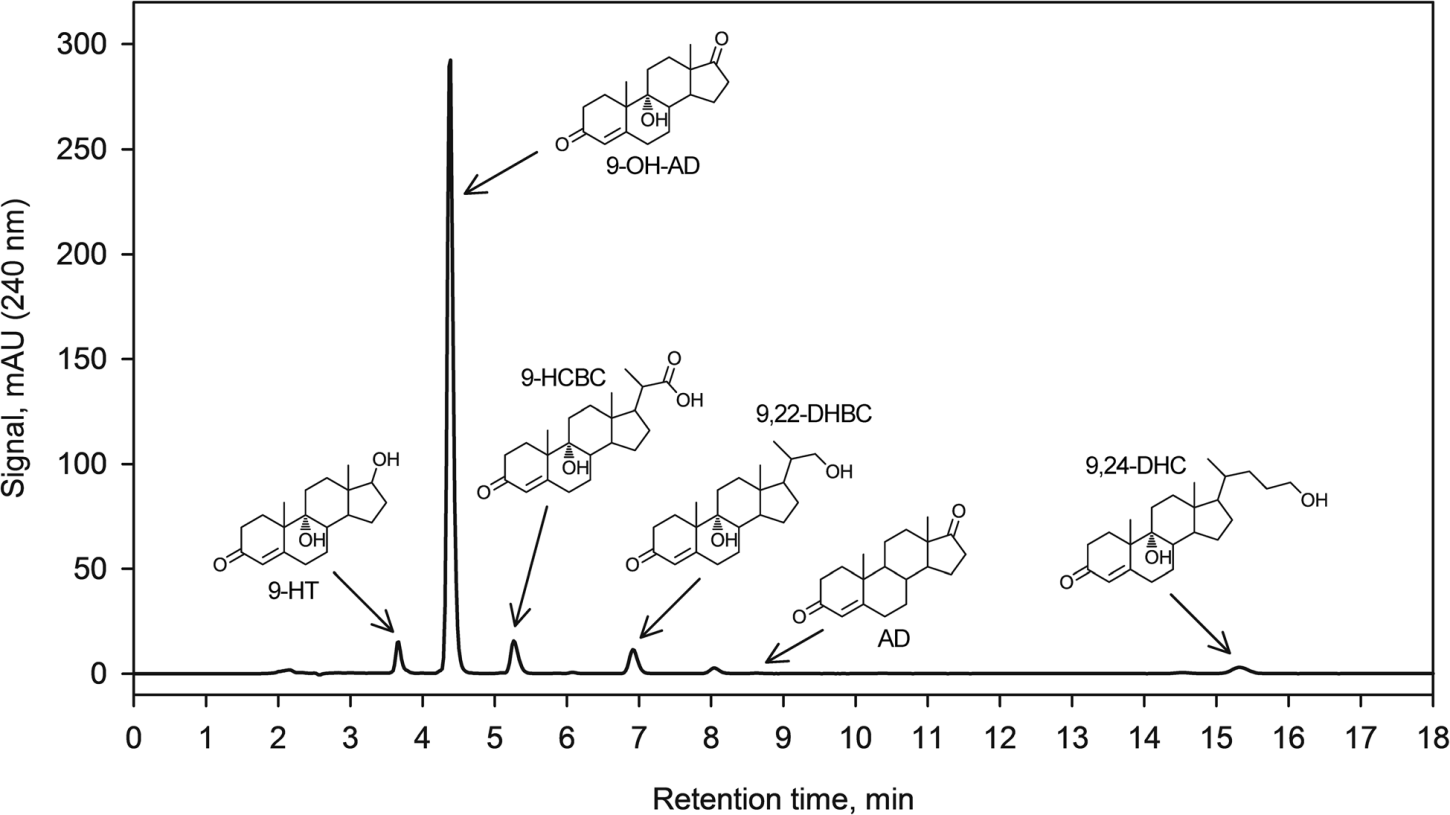
HPLC profile and structures of major steroid products accumulated by *Mycobacterium* sp. VKM Ac-1817D in the induction medium as a result of phytosterol bioconversion

Phytosterol as a sole carbon and energy source provided very slow initial growth and bioconversion activity of *Mycobacterium* sp. VKM Ac-1817D (data not shown). To facilitate growth, both the control and induction media were supplemented with glycerol as a co-substrate. The growth curves are given in Fig. 2. The glycerol-grown cells were harvested in the late active growth stage after 18 h when reaching 1.76 ± 0.05 g/l (d.c.w.) In the induction medium, the culture converted phytosterol mainly to 9-OH-AD as a major product (87% relative abundance (r.a.) after 48 h of incubation, mol/mol) and 9-hydroxylated 3-keto-4-ene steroids with partly, or completely degraded side chain: 9-hydroxy-22-carboxy-23,24-bisnorchol-4-ene-3-one (9-HCBС, r.a 6.5%), 9,22-dihydroxy-23,24-bisnorchol-4-en-3-one (9,22-DHBC, r.a. 4.4%), 9,24-dihydroxychol-4-en-3-one (9,24-DHC, r.a. 2.2%) and 9-hydroxy-testosterone (9-HT, r.a. 1.9%). AD was observed at a noticeably smaller level – 0.12% r.a. (Fig. 3).

The harvesting point for the phytosterol-induced cells was decided on the basis of high specific activity of phytosterol oxidation suggesting maximum level of expression of the specific transcripts. During initial 9 h the conversion rate considerably increased and stabilised at the level of 90–100 μmol/l h g (d.c.w.) until depletion of phytosterol after approx. 30 h. The induced culture was harvested for RNA isolation at the age of 18–21 h. The growth at harvesting point corresponded to 1.7–2.3 g/l (d.c.w.), or approx. 2.5 × 10^8^ CFU/ml.

### Transcriptome sequencing

We studied the differential expression of *Myc* 1817 genes during its growth in medium with phytosterol in comparison with the control medium without any steroids. Statistical parameters of the sequencing results are given in Additional file 1.

For the analysis, we selected the genes whose expression increased by no less than 3-fold at a q-value (false discovery rate) ≤ 0.01. In summary, 260 genes were revealed. The full list of these genes is presented in Additional file 2.

**Fig. 4:**
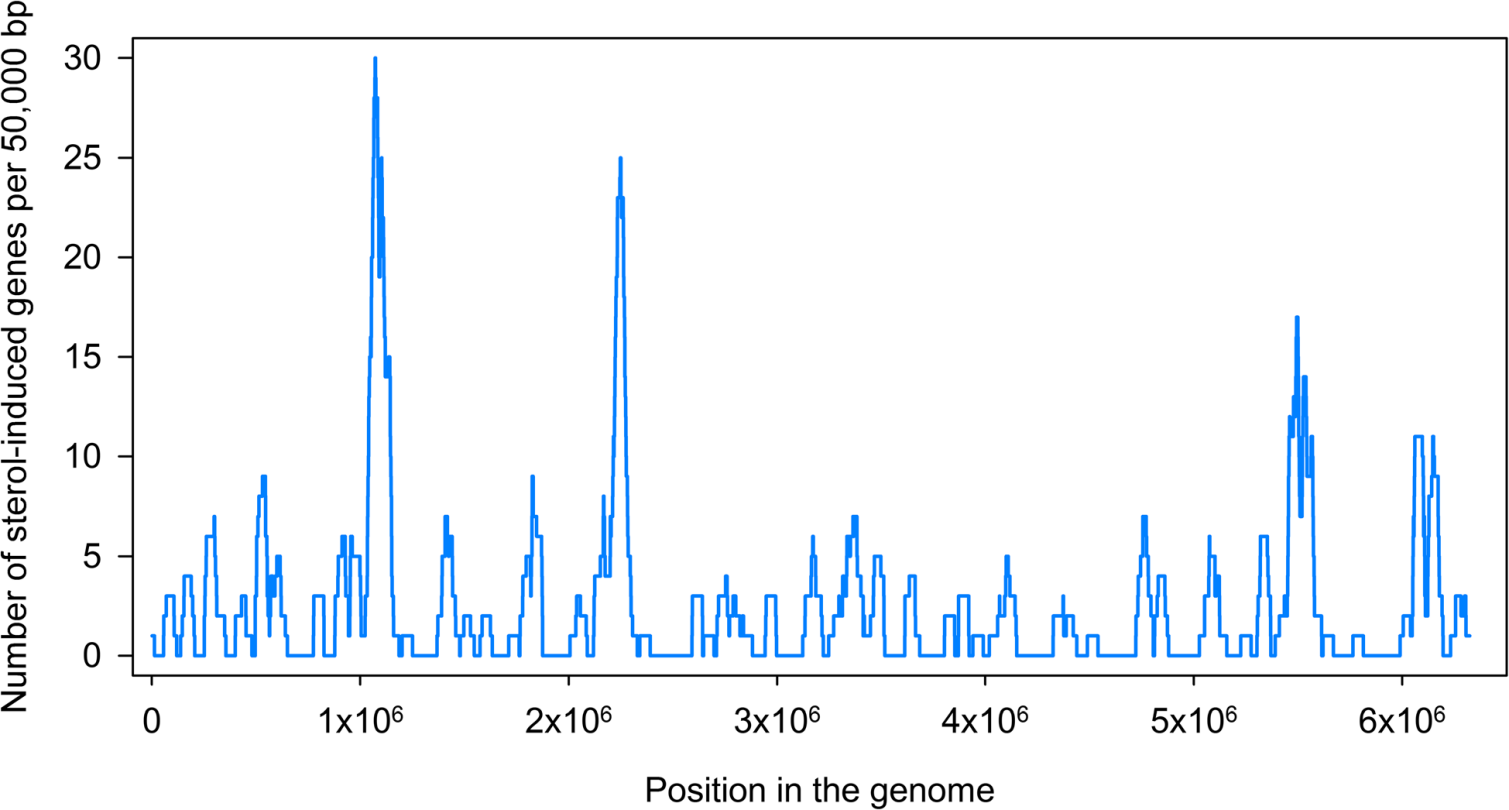
Distribution of phytosterol-induced genes over genome of *Mycobacterium* sp. VKM Ac-1817D. The numbers of sterol-induced genes on a 50.000 bp interval are shown

Sterol-induced genes were distributed irregularly within the *Myc* 1817 genome: they formed some large clusters, as well as a number of relatively small groups. The distribution of the genes which increased their expression in the presence of phytosterol is shown in Fig. 4.

### Real-time PCR

For the expression analysis with real-time PCR, the following single-copy genes with known functions were chosen: (i) *ltp3* and *fadE29*/*chsE2* related to cholesterol side-chain cleavage [17, 20]; (ii) *kstD* and *kshB* encoding the enzymes accounted for the key steps of steroid core degradation [22, 32]; (iii) *fadD3* and *kstR2* that belongs to KstR2-regulon and related to the C/D ring degradation pathway [35]. The three housekeeping genes *rpoB*, *rpoD* and *ftsQ* known for insignificant expression deviations at the transcriptomic studies [45] were used as the reference genes. The results of qRT-PCR qualitatively coincided with the transcriptomic data. The expression of all six genes significantly increased as outlined below from both the transcriptomic and qRT-PCR results (Table 1). Both methods showed similar quantitative results for 4 genes, while the difference was 2-fold for *fadE29*/*chsE2* and the qRT-PCR indicated 5.9-fold higher up-regulation for *kstR2*. The latter may be attributed to the rather unstable expression of the KstR2-regulon genes under the induction conditions, since its putative effector is HIP, but not the early sterol intermediates.

**Table 1:** Phytosterol inducible expression changes of 6 sterol catabolism genes with qRT-PCR data

Gene name	Expression increasing, transcriptomic data	Expression increasing, qRT-PCR data
*fadE29*/*chsE2* (*G155_26165*)	76	152
*ltp3* (*G155_0479*)	97	82
*kshB* (*G155*_*26490*)	40	40
*kstD* (*G155*_*04625*)	61	65
*fadD3* (*G155*_*26340*)	10	13
*kstR2* (*G155*_*26275*)	16	95

### Identification of transcriptional factors binding sites

Putative KstR binding sites were detected for 57 operons including 43 operons with the genes whose expression increased in the presence of phytosterol. Most of the genes related to steroid catabolism were the parts of these operons.

Putative KstR2 binding sites were detected for 8 operons. The genes in all of them were up-regulated on phytosterol. Along with 15 orthologues of the genes putatively involved in the rings C/D degradation [36], we found also additional inducible operon which included the gene of amino hydrolase family *G155_10290* with a putative KstR2-binding site in the promoter (Additional file 3). However, this site was found in only one of the two used programs (UGENE), while the score of this site only slightly exceeds the threshold value.

**Fig. 5:**
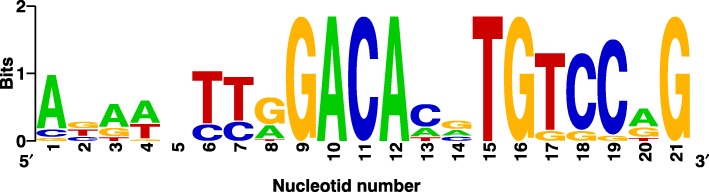
Sequence of motif X, putative binding site of candidate transcription factor *G155_05115*

**Fig. 6:**
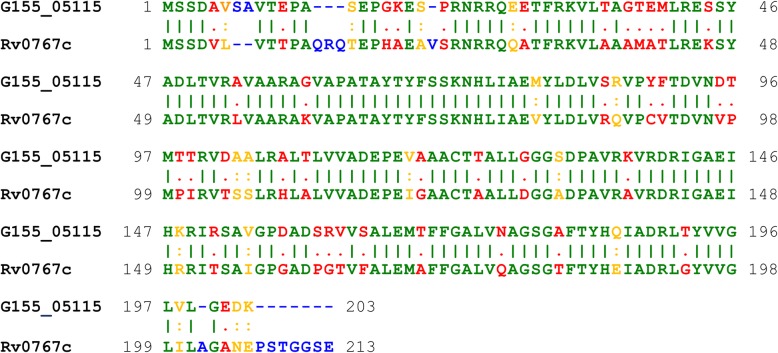
Sequence alignment of the transcription factor G155_05115 from *Mycobacterium* sp. VKM Ac-1817D and its orthologue Rv0767c from *Mycobacterium tuberculosis* H37Rv. Vertical lines represent matching amino acids (green), colons represent similar amino acids (yellow), and dots represent dissimilar (red); gaps/insertions are blue. Different amino acids are called similar if their score in the BLOSUM62 matrix is positive

Among the genes over-expressed in the presence of phytosterol, we found several dozen genes without binding sites of KstR and KstR2. Among them, some orthologues of the known steroid catabolism genes were identified. In the promoters of these genes, a regularly occurring palindromic motif (motif X) was observed (Fig. 5). This motif was very similar to the binding site of *M. tuberculosis* transcription factor Rv0767c [46] with a Q-value of a similarity of 2 × 10^− 10^ (Fig. 6). This means that the similarity was most likely not accidental. The orthologue of *Rv0767c* in *Myc* 1817 is the TetR-family transcription regulator gene *G155_05115*. There was also a sequence that corresponded to the motif X near the start codon of *G155_05115*. This may be a sign of autoregulation, further suggesting that the motif X indeed belongs to this transcription factor.

We identified 14 operons which adjacent with the motif X and presumably regulated with *G155_05115* (Additional file 3). The expression of the genes in 12 of these operons in response to phytosterol increased more than 3-fold, and one operon (*G155_19665*—*G155_19675*) raised expression 2.7-fold, on average. The operons with an adjacent site that is corresponding to motif X included the genes of putative steroid Δ-isomerase (*G155_05080*), some cytochromes, alcohol and aldehyde dehydrogenases, short chain dehydrogenases and TetR-family transcriptional regulators: *G155_05115* itself, *G155_10530*, and *G155_29025*. Among the genes putatively encoded cytochromes, four different genes (namely, *G155_05095*, *G155_05100*, *G155_05110* and *G155_26175*) were presumably coded for cytochrome P450-monooxygenases. Interestingly, that in two cases the sites that corresponded to the motif X were found together with KstR binding sites. For example, the binding sites for both KstR and G155_05115 were found near the promoter of the gene *G155_26175*. Probably, the enzymes which are putatively under regulation of the protein encoded by *G155_05115* with motif X may play role in steroid catabolism.

### Steroid catabolism genes up-regulated in the presence of phytosterol

Among the phytosterol-inducible genes of *Myc* 1817, genes were found whose orthologues were proved, or assumed, to have a function in sterol catabolism (Table 2). These are the genes putatively encoding the side chain oxidation enzymes including starting oxygenases, as well as the genes involved in β-oxidation (encoded acyl-CoA-synthases, enoyl-CoA-hydratases, acyl-CoA-dehydrogenases, aldol lyases, acetyl-CoA-acetyltransferases and homologs of *igr*-operon genes), the genes related to steroid core destruction, uptake of steroids (*mce4*-operon) and the known regulators of steroid catabolism (Table 2) [2, 9, 40, 41]. The genes related to the side chain oxidation significantly (50–136-fold) increased their expression (Table 2).

**Table 2:** Phytosterol inducible expression changes of known sterol catabolism genes

Gene name	Locus	Expression increasing	Function of ortholog	References
*ltp3*	*G155_04795*	96.89	Aldol lyase	[17]
*ltp4*	*G155_04800*	96.38	Aldol lyase	[17]
*echA19*	*G155_04835*	68.59	Enoyl-CoA hydratase	[2]
*fadD19*	*G155_04840*	97.53	Long-chain fatty-acid-CoA ligase	[47]
*fadD17*	*G155_04880*	81.64	Long-chain fatty-acid-CoA ligase	[18]
*chsE5*(*fadE27*)	*G155_04885*	74.14	Acyl-CoA dehydrogenase	[48]
*chsE4*(*fadE26*)	*G155_04890*	89.21	Acyl-CoA dehydrogenase	[48]
*ltp2*	*G155_26150*	69.08	Putative thiolase	[49]
*chsH1*	*G155_26155*	66.32	Steroid enoyl-CoA hydratase	[50]
*chsH2*	*G155_26160*	62.40	Steroid enoyl-CoA hydratase	[50]
*chsE2*(*fadE29*)	*G155_26165*	76.45	Acyl-CoA dehydrogenase	[20]
*chsE1*(*fadE28*)	*G155_26170*	58.19	Acyl-CoA dehydrogenase	[20]
*cyp125*	*G155_26175*	107.58	C26-steroid monooxygenase	[14]
*fadA5*	*G155_26180*	136.07	Acetyl-CoA acetyltransferase	[19]
*chsE3*(*fadE34*)	*G155_26510*	50.22	Acyl-CoA dehydrogenase	[51]
*kstD*	*G155_04625*	61.43	3-oxosteroid 1-dehydrogenase	[52]
*4kstD*	G155_08770	10.09	3-oxosteroid 1-dehydrogenase	[53]
*hsaE*	*G155_04630*	11.57	2-keto-4-pentenoate hydratase	[25]
*hsaG*	*G155_04635*	9.95	Acetaldehyde dehydrogenase	[25]
*hsaF*	*G155_04640*	7.13	4-hydroxy-2-oxovalerate aldolase	[25]
*kshA_1*	*G155_04755*	55.51	3-ketosteroid-9-alpha-hydroxylase oxygenase subunit	[21]
*kshA_2*	*G155_24375*	24.58	3-ketosteroid-9-alpha-hydroxylase oxygenase subunit	[21]
*hsaB*	*G155_26470*	26.88	Monooxygenase component B	[26]
*hsaC*	*G155_26475*	26.18	2,3-dihydroxybiphenyl 1,2-dioxygenase	
*hsaD*	*G155_26480*	32.34	2-hydroxy-6-oxo-6-phenylhexa-2,4-dienoate hydrolase	[27]
*hsaA*	*G155_26485*	34.01	Monooxygenase component A	[26]
*kshB*	*G155_26490*	39.90	3-ketosteroid-9-alpha-hydroxylase reductase subunit	[21]
*hsd*	G155_22700	83.36	3-hydroxysteroid dehydrogenase	[54]
*Hsd4A*	G155_04900	11.12	17β- hydroxysteroid dehydrogenase	[55]
*echA20*	*G155_26220*	9.82	Putative enoyl-CoA hydratase	[38]
*ipdA*	*G155_26225*	8.61	Putative CoA-transferase subunit alpha	[38]
*ipdB*	*G155_26230*	8.35	Putative CoA-transferase subunit beta	[38]
*fadA6*	*G155_26270*	16.55	3-ketoacyl-CoA thiolase, acetyl-CoA acetyltransferase	[38]
*fadE30*	*G155_26335*	10.49	Acyl-CoA dehydrogenase	[38]
*fadD3*	*G155_26340*	9.84	Fatty-acid-CoA ligase	[38]
*fadE31*	*G155_26345*	8.60	Acyl-CoA dehydrogenase	[38]
*fadE32*	*G155_26350*	7.51	Acyl-CoA dehydrogenase	[38]
*fadE33*	*G155_26355*	8.45	Acyl-CoA dehydrogenase	[38]
*kstR*	*G155_26515*	13.17	Transcriptional regulator, TetR family	[28]
*kstR2*	*G155_26275*	15.81	Transcriptional regulator, TetR family	[36]
*yrbEA*	*G155_04905*	3.58	Conserved hypothetical integral membrane protein	[56]
*yrbEB*	*G155_04910*	3.71	Conserved hypothetical integral membrane protein	[56]
*mceA*	*G155_04915*	3.21	MCE-family protein	[56]
*mceB*	*G155_04920*	3.63	MCE-family protein	[56]
*mceC*	*G155_04925*	3.67	MCE-family protein	[56]
*mceD*	*G155_04930*	3.40	MCE-family protein	[56]
*mceE*	*G155_04935*	3.87	MCE-family protein	[56]
*mceF*	*G155_04940*	3.17	MCE-family protein	[56]
*mas4B*	*G155_04950*	3.07	MCE-associated protein	[56]

As described above, Ksh and KstD are the key enzymes of the rings A/B degradation. In our previous study, five genes homologous to oxygenase subunit of 3-ketosteroid-9α-hydroxylase (*kshA*) were revealed in *Myc* 1817 [5]. In this work, we found that two of them are phytosterol-induced: the expression of *kshA_1* (*G155_04755*) and *kshA_2* (*G155_24375*) was increased by 55-fold and 25-fold, respectively (Table 2).

KshA_1 is similar to KshA of *M. smegmatis* (*MSMEG_5925*) by 84%, and to KshA1N of *M. neoaurum* ATCC 25795 — by 85%. KshA1N was shown to express activity towards different 3-oxosteroids including 23,24-bisnorchol-1,4-diene-22-oic acid (1,4-BNC), 4-cholestene-3-one (cholesterone), AD, hydrocortisone, 4-pregnene-3,20-dione (progesterone) and others [30]. KshA_2 showed similarity to MSMEG_5925 by 67%, and KshA1N from *M. neoaurum* ATCC 25795 — by 65%. At the same time, only one of two homologous *kshB* genes of Ksh reductase subunit, namely *kshB_1* (*G155_26490*) was found to be phytosterol-induced: its expression increased 40-fold in response to phytosterol (Table 2).

Among the five putative *kstD* genes encoding 3-ketosteroid Δ^1^-dehydrogenases, only *kstD_1* (*G155_04625*) was over-expressed (in 61-fold) in the presence of phytosterol (Table 2). Expression of the gene *G155_08770* increased in 10 times in the presence of phytosterol. This gene is of 99% similarity to the putative 3-oxo-5α-steroid Δ^4^-dehydrogenase (Δ4(5α)-KSTD) XA26_17580 annotated earlier in *Mycobacterium fortuitum* CT6 genome (CP011269.1) [57]. It is worth noting that *G155_08770* does not show any similarity with the known genes of Δ4(5α)-KSTDs whose protein function has been experimentally confirmed, such as *ro05698* of *Rhodococcus jostii* RHA1 and *Rv1817* of *M. tuberculosis* [53].

The expression of orthologues of the genes whose products are involved in further A/B ring degradation, i.e. *hsaABCD, hsaEFG* and others, increased in 7–34 times (Table 2). Cholesterol oxidases have not been identified among the phytosterol-induced genes, but the *hsd* gene (*G155_22700*) encoding 3β-hydroxysteroid dehydrogenase was over-expressed in the presence of phytosterol by 83-times. The genes of the only one of at least nine *mce*-operons were slightly (in 3–4 times) up-regulated on phytosterol. This operon is similar to the *mce4* operon of *M. smegmatis* mc^2^155 that takes part in sterol transport [56]. Interestingly, the expression of genes of the ATPases associated with the *mce*-operons (G155_06965 and G155_02045) was not increased in response to phytosterol in *Myc* 1817.

Among phytosterol-inducible genes with putative KstR binding sites in *Myc* 1817, the genes of short chain dehydrogenases, 3α(20β)-hydroxysteroid dehydrogenase (*G155_16070)*, aldehyde- and alcohol dehydrogenases, 2 hydride-transferase fragments and many orthologous genes related to fatty acid β-oxidation were revealed (Additional file 3). In *M. smegmatis*, the expression of many orthologues was shown to be increased, e.g., alcohol dehydrogenase *G155_00805*, acyltransferase *G155_01310*, aldehyde dehydrogenase *G155_01350*, 2-nitropropane dioxygenase *G155_16090*, acyl-CoA ligase *fadD8 G155_25385*, and thiolase *G155_17455* [9]. Their products possibly take part in side chain cleavage, or another parts of the phytosterol degradation pathway.

Genes related to the C/D ring degradation pathway that controlled by KstR2 were also up-regulated in phytosterol. In particular, the genes involved in the C/D ring degradation (*echA20, ipdA, ipdB, fadA6, fadD3, fadE30, fadE31, fadE32, fadE33)* showed increased expression by 7–16 times, with an average of 10 times (Table 2).

### Mutations in KshAs, KshB and KstD_1

In order to evaluate whether the capability of *Myc* 1817 to accumulate 9-OH-AD as a major product from phytosterol is associated with malfunctioning of any proteins, we estimated degrees of conservation of the key enzymes of the A/B ring degradation. Amino acid substitutions in evolutionarily conserved positions of KstD_1, KshA, and KshB were evaluated. The known phytosterol degrading strain *M. smegmatis* mc^2^ 155 was used for comparison (NCBI Reference Sequence: NC_008596.1). The results are presented in Table 3.

**Table 3:** Number of amino acid substitution in conservative and non-conservative positions of KshA, KshB and KstD proteins of strain *Myc* 1817 and *M. smegmatis* mc^2^ 155

Protein	Degree of conservation	1	2	3	4	5	6	7	8	9
KshA	KshA_1 (G155_04755)	2	6	9	7	9	14	6	7	48
	KshA_2 (G155_24375)	1	8	5	11	11	11	10	7	65
	KshA_1 (MSMEG_5925)	2	5	3	7	14	7	11	8	44
	KshA_2 (MSMEG_2870)	0	2	3	3	11	3	8	11	40
KshB	KshB (G155_26490)	1	6	8	12	10	11	6	4	36
	(MSMEG_6039)	1	6	11	9	11	8	10	3	32
KstD	KstD (G155_04625)	5	7	7	16	15	12	6	7	55
	KstD (MSMEG_5941)	2	2	15	11	12	10	13	6	52

The amount of substitutions in the most conserved positions (rank 1) in KshA_1 and KshB in *Myc* 1817 was *on a par* similar with that in *M. smegmatis* mc^2^ 155. Of particular note, KshA_2 in *M. smegmatis* mc^2^ 155 has fewer changes in the conservative positions than its orthologue in *Myc* 1817. The greater number of substitutions in the most conserved positions was observed in KstD_1 of *Myc* 1817 as compared with the orthologous KstD in *M. smegmatis* mc^2^ 155 (5 and 2, respectively) (Table 3).

## Discussion

**Fig. 7:**
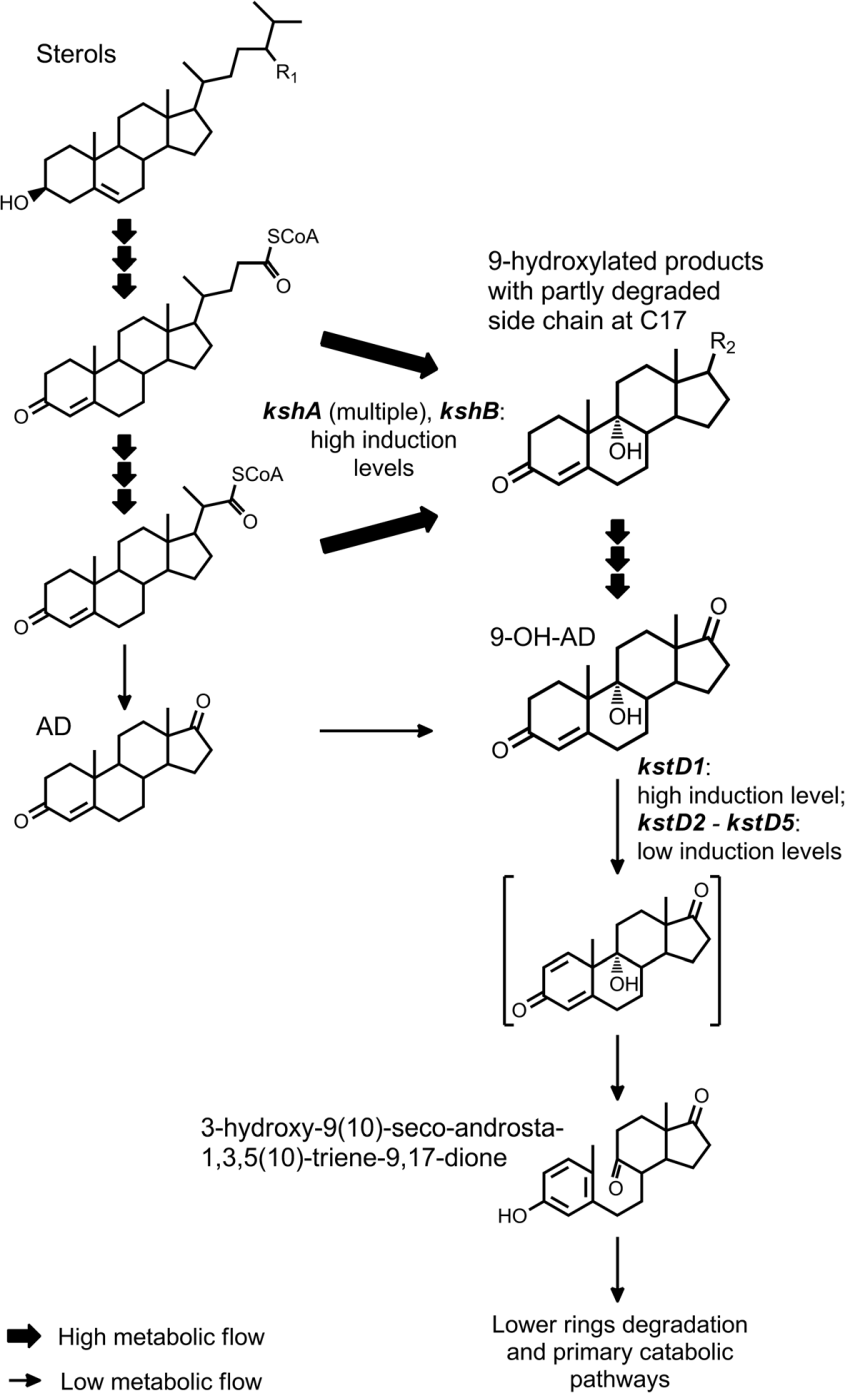
Sterol catabolism and gene expression peculiarities in *Mycobacterium* sp. VKM Ac-1817D. R_1_ – H, CH_3_ or C_2_H_5_ (various sterols); R_2_ – partly degraded side chains. The thickness of arrows reflects suggested relative level of the catabolic activity

The saprotrophic, fast growing native strain *Myc* 1817 is of industrial interest due to its ability to fully transform phytosterol at the high loads with forming 9-OH-AD as a major product [58]. Steroid metabolite profiling of *Myc* 1817D grown in the presence of phytosterol showed the accumulation of 9α-hydroxylated intermediates with a partially oxidised side chain, thus confirming that 9α-hydroxylation precedes full side chain degradation by the strain (Fig. 7). The simultaneous presence of 9α-hydroxylated products with C_3_ and C_5_ side chain in the form of carboxylic acid (9-HCBС) and alcohols (9,22-DHBC, 9,24-DHC) may indicate that its metabolic pathway differs from those known for *M. smegmatis* mc^2^ 155 [9, 59] and *M. neoaurum* ATCC25795 [55], but seems to generally correspond to that suggested for *M. tuberculosis* [21], and some other *Mycobacterium* strains [23, 24, 33].

It had been postulated that accumulation of C_22_– steroids, such as 4-BNC, or 1,4-BNC with some mycobacteria occurs due to the absence, or inefficient 9α-hydroxylation [59]. Accumulation of the corresponding 9α-hydroxyderivatives at the phytosterol conversion with *Myc* 1817D allows to suggest that 9α-hydroxylation is rather not so critical for the elimination of the last isopropyl group of sterol side chain.

Differential transcriptome analysis showed 260 genes that were up-regulated in response to phytosterol in *Myc* 1817, including numerous genes of steroid catabolism. A suite of phytosterol-induced genes related to side-chain, A/B ring and C/D ring degradation was generally similar to that in other phytosterol/cholesterol digesting mycobacteria [2, 9, 40]. Interestingly, the genes which putatively encoded ChOs did not show increased expression in response to phytosterol, while *hsd* (*G155_22700*) was significantly (83 times) up-regulated. This corresponds to the literature data - a similar pattern is observed in *M. smegmatis* [9]. Insignificant up-regulation of *hsd4A* homolog whose product may play a role as 17β-hydroxysteroid dehydrogenase, or β-hydroxyacyl-CoA-dehydrogenase [55] is generally coincided with small amounts of 9-HCBС and 9,22-DHBC observed among the metabolites at phytosterol conversion with *Myc* 1817, and allows to propose that Hsd4A may be active towards 9α-hydroxylated steroids.

Along with the genes involved in the side chain degradation and rings A/B oxidation, the genes related to the lower degradation pathway (rings C/D degradation) were also up-regulated on phytosterol. It is clear that the strain has the ability to fully degrade the steroid core. However, relatively high increase in expression of the rings C/D degradation genes was a bit surprising. These genes belong to KstR2-regulon, which is induced with HIP-CoA, − the compound formed after degradation of the rings A and B [37]. Since *Myc* 1817 mainly accumulates 9-OH-AD, the formation of HIP-CoA was expected to occur much slower than in the sterol-degrading strains, thus predicting attenuation of the KstR2-regulon genes induction.

Effective 9-OH-AD accumulation is generally stipulated with high 3-ketosteroid-9α-hydroxylase (Ksh) and low (or fully blocked) 3-ketosteroid-Δ^1^-dehydrogenase (KstD) activities. In *Myc* 1817, two genes of the oxygenase subunit (*kshA_1* and *kshA_2)* and one gene of the reductase subunit (*kshB_1*) of Ksh significantly (in dozens of times) increased their expression in the presence of phytosterol, while among five putative *kstDs,* an only *kstD_1* was phytosterol-inducible. The mutational analysis showed, however, that the product of this gene contained a significant number of nucleotide substitutions in the conserved positions, thus allowing us to propose either the absence, or low levels of activity. Four remained *kstDs* which did not up-regulated on phytosterol may, nevertheless, be expressed constitutively, thus contributing to the observed steroid core oxidation by the strain. Expression increasing of the KstR2-regulon genes probably also occurs through the activity of these 3-ketosteroid-Δ^1^-dehydrogenases.

A new putative transcriptional regulator G155_05115 involved in the control of steroid catabolism was identified in *Myc* 1817, while most of the genes putatively controlled by this factor did not increased their expression in *M. smegmatis* mc^2^ 155 and *R. jostii* RHA1 [2, 9]. In *Myc* 1817, many genes presumably related to the oxidation of the side chain of sterols were among the putative regulon of this factor.

It is known that in addition to the genes involved in the catabolism of cholesterol and sitosterol, actinobacteria usually have clusters of the genes involved in the catabolism of other steroid compounds and, respectively, regulated by other transcription factors. In particular, there is a cluster of catabolism genes of the cholate [60], and the so-called C-19 cluster, whose genes are involved in the catabolism of yet unknown steroid compounds [61].

Because phytosterol contains β-sitosterol and other plant sterols which differ from cholesterol by branched side chains, it is reasonable to assume that the genes putatively regulated by G155_05115 may play a role in the oxidation of the side chains of the plant sterols of the phytosterol. However, there is a significant number of genes whose relationship with the side chain oxidation, or steroid catabolism remains uncertain in the putative regulon of G155_05115. This indicates that the function of G155_05115-regulon apparently is not limited to the oxidation of the sterols side chain.

Generation of the engineered mycobacterial strains producing valuable steroids is often complicated by homolog multiplicity of steroid catabolism genes [13, 47, 52, 62]. Moreover, various genes capable of performing the same function may have different roles in the catabolism of sterols, as demonstrated for *kstDs*, *hsd4A*, *hsd*, *choD* and others [13, 19, 34, 55, 63]. On the other hand, the known steroid core degradation genes present in the genomes of different mycobacterial strains including those having different metabolic blocks and accumulating specific intermediates of phytosterol degradation process. Knowledge of gene expression regulation in response to phytosterol allows a role for the specific homologous genes in sterol catabolism to be predicted.

## Methods

### Materials

Phytosterol was obtained from S&D Chemicals, UK. Androst-4-ene-3,17-dione (AD) was purchased from Steraloids, USA. Statistically methylated β-cyclodextrin (MCD) was purchased from Wacker Chemie, Germany. Androst-4-ene-3,17-dione (AD) was purchased from Steraloids, USA.9-OH-AD and other 9-hydroxylated steroids (9-HCBС, 9,22-DHBC, 9,24-DHC and 9-HT) with a purity of no less than 98% were obtained at the Institute of Biochemistry and Physiology of Microorganisms, Russian Academy of Sciences (IBPM RAS). The suppliers of other reagents are indicated below.

### Microorganism and cultivation

The strain *Mycobacterium* sp. VKM Ac-1817D was obtained from All-Russian Collection of Microorganisms (VKM IBPM RAS) and pre-cultured as described earlier [64]. The strain was cultured in glycerol-mineral (control) medium [64] and the same medium supplemented with 12 mmol phytosterol (induction medium). Both media contained 24.1 mM MCD. Phytosterol powder and MCD were added to the medium before autoclaving; the sterilized media were sonicated during 2 min on an ultrasonic bath (100 W, 35 kHz) and incubated on an orbital shaker (200 rpm, 30 °C) overnight before inoculation. Experiments were carried out in the two independent biological replicates in 750-ml shake flasks containing 100 ml media.

### Growth estimations

The growth was followed gravimetrically [65] due to high cell-to-cell and cell-to-phytosterol aggregation. Briefly, the samples of the cultivation broth were sedimented by centrifugation at 6500×g for 15 min, then the cakes were washed twice with 40 ml of 10% (w/v) aqueous MCD for phytosterol removal and then twice with 40 ml of distilled water. The washed cells were dried at 70 °C. For viable cell counts, broth samples were serially diluted with 1 g/l aqueous Tween 80 under vigorous agitation and plated on the solid minimal medium. The growth experiments were carried out in three replicates.

### Analytical methods

Phytosterol and steroid metabolites were analysed as described earlier [65] by isocratic reversed-phase HPLC using Waters Symmetry (USA) 250 mm × 4.6 mm (5 μm) column at 50 °C and flow rate 1 ml/min. For phytosterol analysis, the samples were diluted with a mixture of 2-propanol and acetonitrile (45:50, v/v); the analysis was performed using 2-propanol:acetonitrile:deionised water (45:50:5, v/v) as a mobile phase with detection at 200 nm. Total areas of peaks relevant to plant sterols (6–12 min) were used for phytosterol quantification.

For steroid metabolites analysis, the samples were diluted 1:50 with 50% (v/v) aqueous acetonitrile; the analysis was performed using acetonitrile:water:acetic acid (52:48:0.01, v/v) as a mobile phase with detection at 240 nm.

### Isolation of mRNA

The cells were harvested by centrifuge at 8000×g for 10 min and immediately ground in a porcelain mortar under liquid nitrogen. Total RNA was isolated with Qiagen RNeasy mini kit (Qiagen, Netherlands), DNAse I and Ribo-Zero rRNA Removal Kit (Epicentre, USA) according to the protocols of the suppliers.

### High-throughput sequencing

We used TruSeq RNA Sample prep kit v.2 (Illumina, USA) for sample preparation of mRNA for high-throughput sequencing. Sequencing was performed with HiSeq 2000 (50-nucleotide single-read run) according to the protocols of the manufacturer (Illumina, USA).

### Accession numbers

The reads have been deposited in NCBI Sequence Read Archive (SRA) under the accessions numbers: SAMN05941438, and SAMN05941440.

### Transcriptome analysis

For reads mapping and analysis of differential expression the Rockhopper 2.03 have been used [66]. A gene was considered differentially expressed between the conditions with and without phytosterol, if its expression increased, or decreased more than threefold with q-value less, or equal to 0.01.

### Genome annotation

The genome was annotated with RAST (http://rast.theseed.org/FIG/rast.cgi) and PGAP [67]. To find putative genes encoding the enzymes of steroid catabolism and analyse orthologous relations between the genes of *Myc* 1817 and other actinobacteria, we used orthogroups between the genes of *Myc* 1817 and other species of actinobacteria including their plasmids that were constructed by OrthoMCL 2.09 with the OrthoMCL inflation parameter of 1.5, all other parameters were set to the default values.

The operons with the found genes were identified with the internet-service FgenesB (http://linux1.softberry.com/berry.phtml?topic=fgenesb&group=programs&subgroup=gfindb), and with Rockhopper 2.03 on the base of transcriptomic data [52].

### Analysis of transcription factors and binding sites

In order to find binding sites (BSs) of transcription factors which may regulate steroid metabolism, we analysed regions of 500 bp upstream plus 50 bp downstream with respect to start codons of the genes that changed their expression more than threefold with q-value less, or equal to 0.01. The analysis was performed for the up-regulated and down-regulated genes separately. We searched for the over-represented motifs in these regions using MEME 4.10 [68]. Motifs were allowed to be from 8 to 50 bp long. The 20 top-scoring motifs in a form of position-weight matrices (PWMs) were compared with the known motifs of mycobacterial steroid metabolism regulators KstR [28] and KstR2 [36]. The motifs which corresponded to KstR and KstR2 were determined in the lists obtained by MEME. Then, possible motifs of other putative transcription factors were estimated among the remaining motifs in the lists.

Criteria for a motif to be considered as a putative motif of a transcription factor were as follows: 1) the motif must be statistically significant with E-value ≤10^− 5^; 2) the motif must contain a palindrome (palindromic motifs are typical for bacterial transcription factors; and 3) the motif should not be a simple repeat, such as CGCGCGCG…, which is unusual for bacterial transcription factor binding motifs [69]. A single motif that conformed to these criteria was found in a set of the genes that increased their expression and further indicated as “motif X”.

To compare this motif with the known motifs of the transcription factors from TetR family, we replicated a methodology used in [46] to determine motifs of various TetR-family proteins in mycobacteria. The only change in the methodology was that besides 10 species used in that work, we added *Myc* 1817 and other species of our interest, *M. neoaurum* Ac-1815D, to the analysis. After determining the putative motifs of the transcription factors, motif X was compared with them by TOMTOM [70], which is a special tool for motif comparisons from the MEME suite. Then, to seek for all sites of KstR, KstR2 and the transcription factor of motif X in *Myc* 1817 genome we scanned the regions 500 bp upstream plus 50 bp downstream with respect to the start codons of all genes using KstR, KstR2 motifs and motif X found in the previous step. The scan was performed by a tool FIMO from MEME suite [71]. The sites determined with false discovery rate less, or equal to 0.01 (q-value, estimated by FIMO using Benjamini-Hochberg technique) were considered as putative binding sites. For comparison, we also scanned the same regions with the same position weight matrices by UGENE 1.13.1 [72], considering a site as a putative binding site of a transcription factor if its score is no less than 85% of a maximum possible score for the motif of that factor (85% is the default value in UGENE). The binding sites that were presented in the results obtained with both tools (or in the results obtained with one tool, but located before the up-regulated operons) were used in further analyses. The employment of two tools allowed the number of false-positive predictions to be reduced. Detection of a binding site before a first gene of a putative operon predicted by Rockhopper was considered in the analyses as a sign that this binding site regulates all genes in the operon.

The amino acid sequences of the transcription factor G155_05115 from *Myc* 1817 and its orthologue Rv0767c from *M. tuberculosis* H37Rv (GenBank accession number CCP43514) were aligned by EMBOSS Needle 6.6.0 with the default parameters [73].

### Real time PCR

cDNA synthesis was performed using the MMLV reverse transcription kit (Evrogen, Russian Federation) with 0.5 μg of total RNA in accordance with the manufacturer’s instructions. A real-time PCR was carried out using AriaMx Real-time PCR system (Agilent, USA) with Eva Green I M-439 kit (Syntol, Russian Federation). The nucleotide sequences of the primers used in this study for the target and reference genes are listed in Additional file 4. Each sample was run twice and the experiment was performed in duplicate. The amplification was performed as follows: 95 °C for 5 min (1 cycle), 95 °C for 10 s, and 60 °C for 30 s (40 cycles). Gene expression levels were calculated using the ddCq method [74].

### Search of mutations in *kshA*, *kshB* and *kstD* genes

To evaluate amino acid substitutions in proteins KshA_1, KshA_2, KshB and KstD of *Myc* 1817 and their orthologous proteins in *M. smegmatis* mc^2^ 155, we used ConSurf server [75]. For each protein under analysis, it looks for its homologs in 150 close species, performs multiple alignments of their amino acid sequences, and then estimates amino acid conservation for each position. The positions are ranked by ConSurf from the most conserved (rank 1) to the least conserved (rank 9). The most phylogenetically conserved positions in a protein are likely to be crucial for its functioning.

## Conclusions

High expression levels of the genes related to the sterol side chain degradation and steroid 9α-hydroxylation in combination with possible defects in KstD_1 may contribute to effective 9α-hydroxyandrost-4-ene-3,17-dione accumulation from phytosterol provided by this biotechnologically relevant strain. The TetR-family transcription regulator gene *G155_05115* was presumably associated with the regulation of steroid catabolism.

## Additional files

Additional file 1: **Table S1.** Primary results of high-throughput sequencing: number of reads and percentage of rRNA-reads. (XLSX 7 kb)

Additional file 2: **Table S2.** Phytosterol-induced and phytosterol-repressed genes of *Mycobacterium* sp. VKM Ac-1817D. (XLSX 32 kb)

Additional file 3: **Table S3.** Operons of *Mycobacterium* sp. VKM Ac-1817D with putative binding sites of sterol catabolism transcription regulators. (XLSX 23 kb)

Additional file 4: **Table S4.** Primers for qRT-PCR. (XLSX 7 kb)

## Data Availability

The datasets generated during the current study are available in the NCBI SRA repository: https://www.ncbi.nlm.nih.gov/sra/SRX2310504[accn] https://www.ncbi.nlm.nih.gov/sra/SRX2310503[accn] https://www.ncbi.nlm.nih.gov/sra/SRX2310502[accn] https://www.ncbi.nlm.nih.gov/sra/SRX2310501[accn] https://www.ncbi.nlm.nih.gov/sra/SRX2310500[accn] https://www.ncbi.nlm.nih.gov/sra/SRX2310499[accn] https://www.ncbi.nlm.nih.gov/sra/SRX2310498[accn] https://www.ncbi.nlm.nih.gov/sra/SRX2310497[accn]

## Abbreviations

1,4-BNC: 23,24-bisnorchol-1,4-diene-22-oic acid
3-HSA: 3-hydroxy-9,10-seconandrost-1,3,5(10)-triene-9,17-dione
3-HsDs: 3β-hydroxysteroid dehydrogenases
9,22-DHBC: 9,22-dihydroxy-23,24-bisnorchol-4-en-3-one
9,24-DHC: 9,24-dihydroxychol-4-en-3-one
9-HCBС: 9-hydroxy-22-carboxy-23,24-bisnorchol-4-ene-3-one
9-HT: 9-hydroxy-testosterone
9-OH-AD: 9α-hydroxyandrost-4-ene-3,17-dione
AD: Androst-4-ene-3,17-dione
ADD: Androsta-1,4-diene-3,17-dione
CFU: Colony forming unit
ChOs: Cholesterol oxidases
d.c.w: Dry cells weight
HIP: 3aα-*H*-4α(3-propanoate)-7aβ-methylhexahydro-1,5-indanedione
KstD: 3-ketosteroid-∆^1^-dehydrogenase
MCD: Statistically methylated β-cyclodextrin
r.a: Relative abundance
Δ4(5α)-KSTD: 3-oxo-5α-steroid Δ^4^-dehydrogenase

## References

[1] Brzostek A, Sliwiński T, Rumijowska-Galewicz A, Korycka-Machała M, Dziadek J (2005). Identification and targeted disruption of the gene encoding the main 3-ketosteroid dehydrogenase in *Mycobacterium smegmatis*. Microbiol Read Engl..

[2] Van der Geize R, Yam K, Heuser T, Wilbrink MH, Hara H, Anderton MC, Sim E, Dijkhuizen L, Davies JE, Mohn WW, Eltis LD (2007). A gene cluster encoding cholesterol catabolism in a soil actinomycete provides insight into *Mycobacterium tuberculosis* survival in macrophages. Proc Natl Acad Sci U S A.

[3] Donova MV, Egorova OV (2012). Microbial steroid transformations: current state and prospects. Appl Microbiol Biotechnol.

[4] Holert J, Cardenas E, Bergstrand LH, Zaikova E, Hahn AS, Hallam SJ, Mohn WW. Metagenomes reveal global distribution of bacterial steroid catabolism in natural, engineered, and host environments. MBio. 2018. 10.1128/mBio.02345-17. 10.1128/mBio.02345-17 PMC5790920 29382738

[5] Bragin EY, Shtratnikova VY, Dovbnya DV, Schelkunov MI, Pekov YA, Malakho SG, Egorova OV, Ivashina TV, Sokolov SL, Ashapkin VV, Donova MV (2013). Comparative analysis of genes encoding key steroid core oxidation enzymes in fast-growing *Mycobacterium* spp. strains. J Steroid Biochem Mol Biol.

[6] Ge F, Li W, Chen G, Liu Y, Zhang G, Yong B, Wang Q, Wang N, Huang Z, Li W, Wang J, Wu C, Xie Q, Liu G (2011). Draft genome sequence of *Gordonia neofelifaecis* NRRL B-59395, a cholesterol-degrading actinomycete. J Bacteriol.

[7] Rodríguez-García A, Fernández-Alegre E, Morales A, Sola-Landa A, Lorraine J, Macdonald S, Dovbnya D, Smith MCM, Donova M, Barreiro C (2016). Complete genome sequence of “*Mycobacterium neoaurum*” NRRL B-3805, an androstenedione (AD) producer for industrial biotransformation of sterols. J Biotechnol.

[8] Shtratnikova VY, Schelkunov MI, Fokina VV, Pekov YA, Ivashina T, Donova MV (2016). Genome-wide bioinformatics analysis of steroid metabolism-associated genes in *Nocardioides simplex* VKM ac-2033D. Curr Genet.

[9] Uhía I, Galán B, Kendall SL, Stoker NG, García JL (2012). Cholesterol metabolism in *Mycobacterium smegmatis*: cholesterol pathway. Environ Microbiol Rep.

[10] Bergstrand LH, Cardenas E, Holert J, Van Hamme JD, Mohn WW (2016). Delineation of steroid-degrading microorganisms through comparative genomic analysis. mBio..

[11] Kreit J. Microbial catabolism of sterols: focus on the enzymes that transform the sterol 3β-hydroxy-5-en into 3-keto-4-en. FEMS Microbiol Lett. 2017;364(3). 10.1093/femsle/fnx007. 10.1093/femsle/fnx007 28087615

[12] Yao K, Wang FQ, Zhang HC, Wei DZ (2013). Identification and engineering of cholesterol oxidases involved in the initial step of sterols catabolism in *Mycobacterium neoaurum*. Metab Eng.

[13] Ivashina TV, Nikolayeva VM, Dovbnya DV, Donova MV (2012). Cholesterol oxidase ChoD is not a critical enzyme accounting for oxidation of sterols to 3-keto-4-ene steroids in fast-growing *Mycobacterium* sp. VKM ac-1815D. J Steroid Biochem Mol Biol.

[14] Capyk JK, Kalscheuer R, Stewart GR, Liu J, Kwon H, Zhao R, Okamoto S, Jacobs WR, Eltis LD, Mohn WW (2009). Mycobacterial cytochrome P450 125 (Cyp125) catalyzes the terminal hydroxylation of C27 steroids. J Biol Chem.

[15] Rosłoniec KZ, Wilbrink MH, Capyk JK, Mohn WW, Ostendorf M, van der Geize R, Dijkhuizen L, Eltis LD (2009). Cytochrome P450 125 (CYP125) catalyses C26-hydroxylation to initiate sterol side-chain degradation in *Rhodococcus jostii* RHA1. Mol Microbiol.

[16] Johnston JB, Ouellet H, de Montellano PRO (2010). Functional redundancy of steroid C26-monooxygenase activity in *Mycobacterium tuberculosis* revealed by biochemical and genetic analyses. J Biol Chem.

[17] Wilbrink MH, van der Geize R, Dijkhuizen L (2012). Molecular characterization of *ltp3* and *ltp4*, essential for C24-branched chain sterol-side-chain degradation in *Rhodococcus rhodochrous* DSM 43269. Microbiology..

[18] Casabon I, Swain K, Crowe AM, Eltis LD, Mohn WW (2014). Actinobacterial acyl coenzyme a synthetases involved in steroid side-chain catabolism. J Bacteriol.

[19] Nesbitt NM, Yang X, Fontán P, Kolesnikova I, Smith I, Sampson NS, Dubnau E (2010). A thiolase of *Mycobacterium tuberculosis* is required for virulence and production of androstenedione and androstadienedione from cholesterol. Infect Immun.

[20] Thomas ST, Sampson NS (2013). *Mycobacterium tuberculosis* utilizes a unique heterotetrameric structure for dehydrogenation of the cholesterol side chain. Biochem Mosc.

[21] Capyk JK, Casabon I, Gruninger R, Strynadka NC, Eltis LD (2011). Activity of 3-ketosteroid 9α-hydroxylase (KshAB) indicates cholesterol side chain and ring degradation occur simultaneously in *Mycobacterium tuberculosis*. J Biol Chem.

[22] Petrusma M, Hessels G, Dijkhuizen L, van der Geize R (2011). Multiplicity of 3-ketosteroid-9α-hydroxylase enzymes in *Rhodococcus rhodochrous* DSM43269 for specific degradation of different classes of steroids. J Bacteriol.

[23] Donova MV, Gulevskaya SA, Dovbnya DV, Puntus IF (2005). *Mycobacterium* sp. mutant strain producing 9alpha-hydroxyandrostenedione from sitosterol. Appl Microbiol Biotechnol.

[24] Yao K, Xu LQ, Wang FQ, Wei DZ (2014). Characterization and engineering of 3-ketosteroid-Δ^1^-dehydrogenase and 3-ketosteroid-9α-hydroxylase in *Mycobacterium neoaurum* ATCC 25795 to produce 9α-hydroxy-4-androstene-3,17-dione through the catabolism of sterols. Metab Eng.

[25] Carere J, McKenna SE, Kimber MS, Seah SYK (2013). Characterization of an aldolase-dehydrogenase complex from the cholesterol degradation pathway of *Mycobacterium tuberculosis*. Biochem Mosc.

[26] Dresen C, Lin LY-C, D’Angelo I, Tocheva EI, Strynadka N, Eltis LD (2010). A Flavin-dependent monooxygenase from *Mycobacterium tuberculosis* involved in cholesterol catabolism. J Biol Chem.

[27] Lack NA, Yam KC, Lowe ED, Horsman GP, Owen RL, Sim E, Eltis LD (2010). Characterization of a carbon-carbon hydrolase from *Mycobacterium tuberculosis* involved in cholesterol metabolism. J Biol Chem.

[28] Kendall SL, Withers M, Soffair CN, Moreland NJ, Gurcha S, Sidders B, Frita R, Ten Bokum A, Besra GS, Lott JS, Stoker NG (2007). A highly conserved transcriptional repressor controls a large regulon involved in lipid degradation in *Mycobacterium smegmatis* and *Mycobacterium tuberculosis*. Mol Microbiol.

[29] Dovbnya DV, Egorova OV, Donova MV (2010). Microbial side-chain degradation of ergosterol and its 3-substituted derivatives: a new route for obtaining of deltanoids. Steroids..

[30] Liu H-H, Xu L-Q, Yao K, Xiong L-B, Tao X-Y, Liu M, Wang F-Q, Wei D-Z (2018). Engineered 3-ketosteroid 9α-hydroxylases in *Mycobacterium neoaurum*: an efficient platform for production of steroid drugs. Appl Environ Microbiol.

[31] Szaleniec M, Wojtkiewicz AM, Bernhardt R, Borowski T, Donova M (2018). Bacterial steroid hydroxylases: enzyme classes, their functions and comparison of their catalytic mechanisms. Appl Microbiol Biotechnol.

[32] Capyk JK, D’Angelo I, Strynadka NC, Eltis LD (2009). Characterization of 3-ketosteroid 9α-hydroxylase, a Rieske oxygenase in the cholesterol degradation pathway of *Mycobacterium tuberculosis*. J Biol Chem.

[33] Li Hui, Wang Xiangdong, Zhou Longfei, Ma Yang, Yuan Wanjuan, Zhang Xiaomei, Shi Jinsong, Xu Zhenghong (2018). Enhancing Expression of 3-Ketosteroid-9α-Hydroxylase Oxygenase, an Enzyme with Broad Substrate Range and High Hydroxylation Ability, in Mycobacterium sp. LY-1. Applied Biochemistry and Biotechnology.

[34] Zhang R, Liu X, Wang Y, Han Y, Sun J, Shi J, Zhang B (2018). Identification, function, and application of 3-ketosteroid Δ^1^-dehydrogenase isozymes in *Mycobacterium neoaurum* DSM 1381 for the production of steroidic synthons. Microb Cell Factories.

[35] Casabon I, Zhu S-H, Otani H, Liu J, Mohn WW, Eltis LD (2013). Regulation of the KstR2 regulon of *Mycobacterium tuberculosis* by a cholesterol catabolite. Mol Microbiol.

[36] Kendall SL, Burgess P, Balhana R, Withers M, ten Bokum A, Lott JS, Gao C, Uhia-Castro I, Stoker NG (2010). Cholesterol utilization in mycobacteria is controlled by two TetR-type transcriptional regulators: *kstR* and *kstR2*. Microbiology..

[37] Casabon I, Crowe AM, Liu J, Eltis LD (2013). FadD3 is an acyl-CoA synthetase that initiates catabolism of cholesterol rings C and D in actinobacteria: role of FadD3 in cholesterol catabolism. Mol Microbiol.

[38] Crowe AM, Casabon I, Brown KL, Liu J, Lian J, Rogalski JC, Hurst TE, Snieckus V, Foster LJ, Eltisa LD. Catabolism of the last two steroid rings in *Mycobacterium tuberculosis* and other bacteria. MBio. 2017. 10.1128/mBio.00321-17. 10.1128/mBio.00321-17 PMC5380842 28377529

[39] van der Geize R, Grommen AWF, Hessels GI, Jacobs AC, Dijkhuizen L (2011). The steroid catabolic pathway of the intracellular pathogen *Rhodococcus equi* is important for pathogenesis and a target for vaccine development. PLoS Pathog.

[40] Li Q, Ge F, Tan Y, Zhang G, Li W (2016). Genome-wide transcriptome profiling of *Mycobacterium smegmatis* mc^2^ 155 cultivated in minimal media supplemented with cholesterol, androstenedione or glycerol. Int J Mol Sci.

[41] Liu M, Xiong LB, Tao X, Liu QH, Wang FQ, Wei DZ (2018). Integrated transcriptome and proteome studies reveal the underlying mechanisms for sterol catabolism and steroid production in *Mycobacterium neoaurum*. J Agric Food Chem.

[42] Gupta RS, Lo B, Son J (2018). Phylogenomics and comparative genomic studies robustly support division of the genus *Mycobacterium* into an emended genus *Mycobacterium* and four novel genera. Front Microbiol.

[43] Sukhodolskaya GV, Nikolayeva VM, Khomutov SM, Donova MV (2007). Steroid-1-dehydrogenase of *Mycobacterium* sp. VKM ac-1817D strain producing 9alpha-hydroxy-androst-4-ene-3,17-dione from sitosterol. Appl Microbiol Biotechnol.

[44] Shtratnikova VY, Schelkunov MI, Dovbnya DV, Pekov YA, Bragin EY, Ashapkin VV, Donova MV (2015). Complete genome sequence of *Mycobacterium* sp. strain VKM ac-1817D, capable of producing 9α-hydroxy-androst-4-ene-3,17-dione from phytosterol. Genome Announc.

[45] Rocha DJP, Santos CS, Pacheco LGC (2015). Bacterial reference genes for gene expression studies by RT-qPCR: survey and analysis. Antonie Van Leeuwenhoek.

[46] Balhana RJC, Singla A, Sikder MH, Withers M, Kendall SL (2015). Global analyses of TetR family transcriptional regulators in mycobacteria indicates conservation across species and diversity in regulated functions. BMC Genomics.

[47] Wilbrink MH, Petrusma M, Dijkhuizen L, van der Geize R (2011). FadD19 of *Rhodococcus rhodochrous* DSM43269, a steroid-coenzyme a ligase essential for degradation of C-24 branched sterol side chains. Appl Environ Microbiol.

[48] Wipperman MF, Yang M, Thomas ST, Sampson NS (2013). Shrinking the FadE proteome of *Mycobacterium tuberculosis*: insights into cholesterol metabolism through identification of an α_2_β_2_ heterotetrameric acyl coenzyme a dehydrogenase family. J Bacteriol.

[49] Gilbert S, Hood L, Seah SYK. Characterization of an aldolase involved in cholesterol side chain degradation in *Mycobacterium tuberculosis*. J Bacteriol. 2017;200. 10.1128/JB.00512-17. 10.1128/JB.00512-17 PMC5738731 29109182

[50] Yang M, Guja KE, Thomas ST, Garcia-Diaz M, Sampson NS (2014). A distinct MaoC-like enoyl-CoA hydratase architecture mediates cholesterol catabolism in *Mycobacterium tuberculosis*. ACS Chem Biol.

[51] Yang M, Lu R, Guja KE, Wipperman MF, St Clair JR, AC B, Garcia-Diaz M, Sampson NS (2015). Unraveling cholesterol catabolism in *Mycobacterium tuberculosis*: ChsE4-ChsE5 α_2_β_2_ acyl-CoA dehydrogenase initiates β-oxidation of 3-oxo-cholest-4-en-26-oyl CoA. ACS Infect Dis.

[52] van der Geize R, Hessels GI, Dijkhuizen L (2002). Molecular and functional characterization of the kstD2 gene of *Rhodococcus erythropolis* SQ1 encoding a second 3-ketosteroid delta(1)-dehydrogenase isoenzyme. Microbiol Read Engl.

[53] van Oosterwijk N, Knol J, Dijkhuizen L, van der Geize R, Dijkstra BW (2012). Structure and catalytic mechanism of 3-ketosteroid-Delta4-(5α)-dehydrogenase from *Rhodococcus jostii* RHA1 genome. J Biol Chem.

[54] Drzyzga O, Fernandez de las Heras L, Morales V, Navarro Llorens JM, Perera J (2011). Cholesterol degradation by *Gordonia cholesterolivorans*. Appl Environ Microbiol.

[55] Xu LQ, Liu YJ, Yao K, Liu HH, Tao XY, Wang FQ, Wei DZ (2016). Unraveling and engineering the production of 23,24-bisnorcholenic steroids in sterol metabolism. Sci Rep.

[56] García-Fernández J, Papavinasasundaram K, Galán B, Sassetti CM, García JL (2017). Molecular and functional analysis of the mce4 operon in *Mycobacterium smegmatis*. Environ Microbiol.

[57] Costa KC, Bergkessel M, Saunders S, Korlach J, Newman DK (2015). Enzymatic degradation of phenazines can generate energy and protect sensitive organisms from toxicity. MBio..

[58] Donova MV, Dovbnya DV, Kalinichenko AN, Vagabova LM, Arinbasarova AY, Morozova ZV, Koshcheyenko KA (1997). Method of 9-alpha-hydroxyandrost-4-ene-3,17-dione preparing.

[59] Galán B, Uhía I, García-Fernández E, Martínez I, Bahíllo E, de la Fuente JL, Barredo JL, Fernández-Cabezón L, García JL (2017). *Mycobacterium smegmatis* is a suitable cell factory for the production of steroidic synthons. Microb Biotechnol.

[60] Mohn WW, Wilbrink MH, Casabon I, Stewart GR, Liu J, van der Geize R, Eltis LD (2012). Gene cluster encoding cholate catabolism in *Rhodococcus* spp. J Bacteriol.

[61] Fernandez-Cabezon L, Galan B, Garcia JL (2018). Unravelling a new catabolic pathway of C-19 steroids in *Mycobacterium smegmatis*. Environ Microbiol.

[62] Knol J, Bodewits K, Hessels GI, Dijkhuizen L, van der Geize R (2008). 3-keto-5alpha-steroid Delta(1)-dehydrogenase from *Rhodococcus erythropolis* SQ1 and its orthologue in *Mycobacterium tuberculosis* H37Rv are highly specific enzymes that function in cholesterol catabolism. Biochem J.

[63] García JL, Uhía I, Galán B (2012). Catabolism and biotechnological applications of cholesterol degrading bacteria. Microb Biotechnol.

[64] Donova MV, Nikolayeva VM, Dovbnya DV, Gulevskaya SA, Suzina NE (2007). Methyl-beta-cyclodextrin alters growth, activity and cell envelope features of sterol-transforming mycobacteria. Microbiology..

[65] Shtratnikova VY, Schelkunov MI, Dovbnya DV, Bragin EY, Donova MV (2017). Effect of methyl-β-cyclodextrin on gene expression in microbial conversion of phytosterol. Appl Microbiol Biotechnol.

[66] Tjaden B (2016). *De novo* assembly of bacterial transcriptomes from RNA-seq data. Genome Biol.

[67] Zhao Y, Wu J, Yang J, Sun S, Xiao J, Yu J (2012). PGAP: pan-genomes analysis pipeline. Bioinformatics.

[68] Bailey TL, Elkan C (1994). Fitting a mixture model by expectation maximization to discover motifs in biopolymers. Proc Int Conf Intell Syst Mol Biol.

[69] Rodionov DA (2007). Comparative genomic reconstruction of transcriptional regulatory networks in bacteria. Chem Rev.

[70] Gupta S, Stamatoyannopoulos JA, Bailey TL, Noble WS (2007). Quantifying similarity between motifs. Genome Biol.

[71] Grant CE, Bailey TL, Noble WS (2011). FIMO: scanning for occurrences of a given motif. Bioinforma Oxf Engl.

[72] Okonechnikov K, Golosova O, Fursov M (2012). Unipro UGENE: a unified bioinformatics toolkit. Bioinformatics.

[73] Rice P, Longden I, Bleasby A (2000). EMBOSS: the European molecular biology open software suite. Trends Genet.

[74] Livak KJ, Schmittgen TD (2001). Analysis of relative gene expression data using real-time quantitative PCR and the 2^−ΔΔC^_T_ method. Methods..

[75] Ashkenazy H, Abadi S, Martz E, Chay O, Mayrose I, Pupko T, Ben-Tal N (2016). ConSurf 2016: an improved methodology to estimate and visualize evolutionary conservation in macromolecules. Nucleic Acids Res.

